# Urban water and food security in this century and beyond: Resource-smart cities and residents

**DOI:** 10.1007/s13280-020-01373-1

**Published:** 2020-10-01

**Authors:** Jan-Olof Drangert

**Affiliations:** Department of Water and Environmental Studies, Linköping University, Linköping, Sweden

**Keywords:** Recycled plant nutrients, Recycled urban water, Solid and liquid waste hierarchy, Sustainable access to water and food, Urban water balance, Water-efficient urban infrastructure

## Abstract

The urban world population will increase from 3 to 8.5 thousand million in the 21st century. Cities become hot spots of both demand for water and global food and for disposed used water and nutrients. Sustainability requires that resource flows through our cities are co-managed and connected to agriculture. Reduced use of harmful chemicals in consumer products facilitates treatment to a quality that allows reuse/recycling of water and nutrients. A solid and liquid waste hierarchy can assist in ordering measures. A novel flexible water balance can guide city infrastructure and keep toilet water separate. New water-saving equipment can substantially reduce water use without losing personal comfort. The combination of these new approaches ascertains access to safe urban water, and that recovered nutrients from cities can substitute half of chemical fertilisers needed in food production. Now, thousands of new cities and suburbs provide unique opportunities to develop resource-smart and sustainable flows.

## Introduction

Sustainability requires that we accept the fact that Mother Earth and the embracing atmosphere are limited—despite being immense (Folke et al. 1997). For the first time in human history, the Earth’s system boundary is being crossed (Rockström et al. 2009; Steffen et al. 2015; Zhang et al. 2015). Today, emissions of greenhouse gases, cropland use, and application of nitrogen and phosphorus have exceeded Nature´s capacity to provide physical resources and sinks in a sustainable way (Springmann et al. 2018).

This realisation has helped spark a deepened interest in sustainable management of existing resources. We urgently need to address and consider the whole value chain from exploiting natural resources via production and consumption, to management of so called waste. In this article the focus is on potentials and limits to enhance urban management of water and nutrients for perceived needs.

The last century and a half saw a wasteful use of water and removal of urban organic matter. Sewers were introduced to move excreta and wastewater away from urban areas in order to solve urban sanitary challenges (Melosi 2000). No one opposed measures to reduce human exposure to pathogens, but the loss of the fertilising value in urban waste was initially of great concern (e.g. Hugo 1862). Moreover, there has been a tendency to overestimate Nature’s capacity to accommodate rising volumes of nutrient-rich but contaminated wastewater and sludge in seemingly endless water bodies. The world now witness the consequences with, e.g. eutrophic lakes and dead bottoms of seas (UNEP 2006).

We have also overstated the atmosphere’s capacity to accommodate rising emissions from natural and man-made processes producing greenhouse gases that cause global warming. The water, sanitation and agricultural sectors contribute to this in several ways, for instance through methane releases from decaying organic material (e.g. excreta, bio waste, and sludge), use of fossil energy to transport water and fertilisers, and in particular the use of natural gas to manufacture nitrogen to produce chemical fertilisers. A recycling society could reduce or do away with much of these emissions. A Swedish study estimates that if all N in the toilet water (equals 20% of mineral nitrogen applied each year in Swedish agriculture) is recycled, the climate impact will equal a case where 80% of the used mineral fertilisers were produced without releasing any climate-affecting emissions (Jönsson 2019). However, recirculation requires that households reduce the use of complex chemical products in order to improve the quality of discharged water and nutrients. People may want to get rid of toxic chemicals, but this time a resourceful chemical industry opposes and lobby against restrictions on their activities (ECHA 2007; Kümmerer 2007).

The sustainable development goals for 2030 adopted by the UN General Assembly in 2015 show a shift in focus from rural to urban water and sanitation in order to remain relevant in a rapidly urbanising world (UN 2015). While retaining the goal to ensure access to clean water and proper sanitation for all (Goal 6), the emphasis is on sustainable production and consumption of water and food, and reduced discharges of polluted water, as evidenced in the following excerpts:

- Rapid urbanisation is exerting pressure on fresh water supplies, sewage, the living environment, and public health (Goal 11).
- Water is free from nature but the infrastructure needed to deliver it is expensive (Goal 12).
- Excessive use of water contributes to the global water stress (Goal 12).
- The high density of cities can bring efficiency gains and technological innovation while reducing resource and energy consumption (Goal 11).
- Man is polluting water faster than nature can recycle and purify water in rivers and lakes (Goal 12).
- More than 80% of wastewater resulting from human activities is discharged into rivers or sea without any pollution removal (Goal 6).
- Land degradation, declining soil fertility, unsustainable water use, overfishing and marine environment degradation are all lessening the ability of the natural resource base to supply food (Goal 12).

This article outlines how cities through a systems management approach can contribute to sustainability. The present competition between urban areas and farmland for water and nutrients can be ameliorated by increased interaction between the two sectors. The chosen approach is to consider flows of water, food, wastewater and biowaste between urban areas and farmland as circular flows. Earlier and present water management practices are explored in order to develop sustainable strategies and measures to secure future urban water supply as well as the availability of nutrients for food production while also protecting human health and the environment.

## Methods beyond integrated resources management to secure water and food supplies

The focus is on household water and nutrient flows in urban areas. The system border is defined by flows that are or can be managed by cities. Attention goes to control what will later end up in biowaste, wastewater and sludge, and actions begin where waste originates—a reversal of the main focus on “end-of-pipe” treatment up to now.

**Fig. 1:**
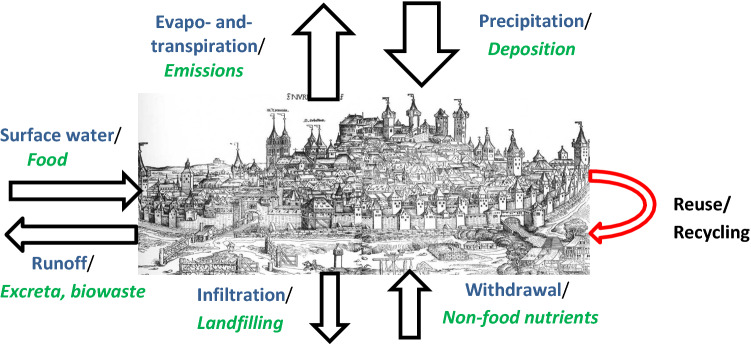
Urban water exchange with atmosphere (precipitation and evapotranspiration), surface water (lakes, rivers, and runoff), ground/soil (infiltration and withdrawal) and reuse/recycling of used water. Nutrient exchanges (*in green italics*) comprise mainly food and non-food nutrients, excreta and biowaste, and reuse/recycling of nutrients

Figure 1 shows the exchanges of water between the atmosphere, soil surface, groundwater, and recirculation of used water. Likewise, exchanges of nutrients take place via flows of food, excreta and other biowaste, deposition from and emissions to the atmosphere, import of non-food nutrients, and recirculation of nutrients.

The European Union *waste hierarchy* provides a helpful systems- and lifecycle-based tool to structure our thinking about usage and flows of water and plant nutrient resources (EU 2008). This hierarchy is extended here to also include liquid waste in order to be able to analyse recovery and recycling of most water and nutrients in urban waste flows. Today, households slowly begin to separate organic waste for compost or biogas, while municipal utilities in most towns and cities manipulate surface water and stormwater, groundwater recharge and withdrawal, and wastewater discharges. Also, efforts emerge to reuse and recycle the content in already used water in, e.g. EU and the U.S. (EC 2015; WEF 2018). A novel partly dynamic *flexible urban water balance* is introduced here to explore ways to address a looming urban water crisis (Drangert and Sharatchandra 2017). The new approach is to combine these two tools in order to simultaneously manage the various water and nutrient flows.

### The extended waste hierarchy

The five steps below make up the “extended waste hierarchy” which can guide the transformation of urban water and sanitation systems to become more sustainable:

Step 1 *Reduce* (a) waste generation and (b) harmful contents in products and flows;
Step 2 *Reuse* the used water and nutrients more or less as they are;
Step 3 *Recycle* the treated wastewater, sludge and biowaste as input to new products (including biogas generation);
Step 4 *Incinerate* biowaste and sludge to reduce its volume and extract the remaining energy content;
Step 5 Safely *landfill* ashes and sludge residues and dispose of water after exhausting the previous steps.

Up to 2014, Steps 5 and 4 were the most common practices for solid waste and wastewater sludge in Europe (Eurostat 2016). A sharp focus on the first three steps—applied to both solid and liquid “wastes”—is needed to sustain water supply and food security. Reduced generation of nutrient-rich waste and enhanced recovery have wide environmental benefits such as cleaner cities and reduced eutrophication of water bodies.

N in recycled organic waste saves energy otherwise needed to manufacture nitrogen N_2_ in mineral fertilisers, often by using natural gas (Zhang et al. 2015; WEF 2018), and not needing fossil energy to remove N in human excreta at the treatment plant. A Swedish study estimates that if all N in the blackwater (equals 20% of mineral nitrogen applied each year in Swedish agriculture) is recycled, the climate impact will equal a case where 80% of the used mineral fertilisers were produced without releasing any climate-affecting emissions (Jönsson 2019). Likewise, by replacing mineral fertilisers with, e.g. phosphorus-rich organic waste, substantial amounts of polluting wastes generated as by-products in the mining and processing of phosphate rock are avoided (Ayres et al. 2001). Thus, replacing mineral fertilisers with local fertilisers derived from slightly processed nutrient-rich solid and liquid wastes achieves both environmental and economic benefits (Jönsson 2019).

### The flexible water balance

**Fig. 2:**
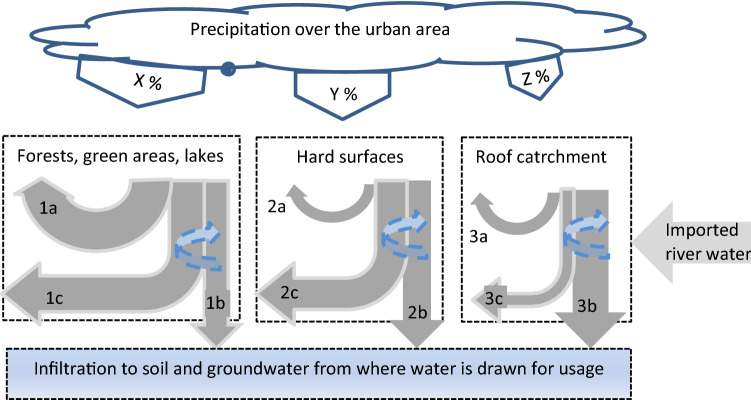
The “*flexible water balance*” with crucial water flows in an urban area. Rain on various surfaces is evaporated to atmosphere (**a**), infiltrated to soil and groundwater (**b**), and run off to water bodies (**c**). The dashed bent arrows represent reuse and recycling

The urban catchment area is divided into three parts: roofs; hard surfaces; and forests, lakes, and farmland (Fig. 2), since these require different reuse/recycling methods. The precipitation during the full year or only the wet or dry season, as an average or within a range, is distributed between the three categories of surfaces and *x*% + *y*% + *z*% = 100%. Municipalities keep data on land area use (km^2^) and on precipitation (mm year^−1^). Estimates of evaporation and evapotranspiration from various surfaces, index a, are used to calculate losses to the atmosphere. Likewise, infiltration to soil and groundwater, index b, and runoff, index c, can be estimated for any city area. In addition, rainwater from the larger catchment area can be imported to the city from an easy access point in a river or lake.

Different stakeholders, sometimes with conflicting interests, are responsible for anticipated management measures to manipulate the flows of rainwater, surface water, groundwater and recycled water (McConville et al. 2015).

In a second phase, some of the recharged groundwater (Fig. 2, bottom) may be withdrawn for renewed household and industrial use without lowering the initial groundwater level. Given that users refrain from seriously polluting water while using it, the treated effluent together with some fresh water can be used again and again. After renewed use this used water is eventually brought back to the groundwater or other storage after some treatment.

Singapore city-state water supply represents an interesting example of harvesting urban rain and wastewater on a large scale (PUB 2014). The 6 million Singaporeans live on an island measuring only 646 km^2^. Two-thirds of the island serves as a water catchment, from where no water flows directly to the ocean. The rainfall on this area is collected through an 8000 km network of drains, canals, rivers, stormwater collection ponds and reservoirs. All water is treated and nanofiltrated to drinking water quality and supplied to the residents and industries in Singapore.

## Research and empirical data on water and nutrient recovery and renewed use

Water and nutrients are available both in situ and further away. The need to extract virgin water and nutrient resources can be minimised by improving system effectiveness and efficiency, without compromising health or comfort. The focus here is on accessing local urban water sources, and returning nutrients in treated organic waste including food waste, excreta and sludge to agriculture.

### Step 1: Reduce waste generation by optimising water and nutrient use efficiency

Step 1 is the most important step in the hierarchy due to its impact on what amount and quality of resources proceed to the various flows. Upstream measures that minimise harmful and unwanted chemical substances in products and materials as well as pathogens in wastes can facilitate treatment of organic waste and wastewater to recyclable quality.

The water sector has been slow to engage in managing demand. Design values in litres per person per day (lpcd) for urban households have only slowly come down in the last decades (Gleick 1993; Suzuki et al. 2010). One possible explanation is that water is viewed as a human right according to United Nations. However, such right still allows water to be priced—and subsidised for the poor. Another explanation is that, unlike electricity, water to apartments is rarely metered and billed according to volume in OECD countries (OECD 1999). Instead, costs are shared between all residents. This hiding of the cost of water discourages prudent use. Apartments could easily be fitted with individual metres for both cold and hot water with a progressive tariff—just like water usage in single-family houses.

The water sector has also been slow to introduce compulsory water-saving fixtures, despite the many water-saving equipment and devices available on the world market (See Table 1) that can substantially reduce water use without loss of personal comfort (Gleick 1993, Tables H24–25; Suzuki et al. 2010). Simultaneously, the volume of wastewater would go down proportionately, and become cheaper to treat. One valid sector argument is that with less water some sewers may get clogged. However, new housing areas can avoid this clogging restriction by installing more decentralised systems with pipes with sufficient slope.

**Table 1:** Various measures by urban households in some countries to reduce, reuse and recycle water in litres per person per day and nutrients as percentage of imported mined P. Sources: Gleick (1993), Suzuki et al. (2010), Svenskt Vatten (2017), Drangert et al. (2018)

Measures to save H_2_O in some rich cities/countries					Measures to save P in EU member states	
	USA ca 1990 Range	USA Least lpcd	Sweden today (L)	Aim in lpcd (L)		EU import (%)
**Step 1: reduce**						
Hygiene eg. shower	20–30 (L per min)	100–150 L 5 min/day	60	− 30	No P in detergents	− 6
Toilet flush	10–30 (L per flush)	75–210 L 7 times/day	30	− 15	No P in additives	− 14
Washing clothes	150–210 (L per load)	35–52 L once/week	15	− 5	Less food waste	− 10
Washing dishes	50–120 (L per load)	17–40 L twice/week	15	− 5	More vegetarian food	− *X*
Prepare food + drink	No measure	20 L	10	0		
Other	No measure	20 L	10	0		
***Subtotal***		***267–492 L***	***140***	− ***55***		**>** − ***30***
**Step 2: reuse**						
Greywater	0	0	0	− 10	Urine	− 31
Blackwater	0	0	0	− 2	Food waste	− 5
***Subtotal***	***0***	***0***	***0***	***− 12***		***− 36***
**Step 3: recycle**						
Greywater	0	0	0	− 35	Faeces	− 16
Blackwater	0	0	0	− 10	Food waste	− 8
***Subtotal***	***0***	***0 ***	***0***	***− 45***		***− 24***

Nutrients can be recovered from urban waste streams, and phosphorus (P) is delved upon in some detail. Processed mined phosphate is used as P-fertiliser, as input in detergents, and increasingly as food and feed additives (van Dijk et al. 2016). The P in detergents can be replaced with harmless zeolite in combination with polycarboxylates, and P in additives can be reduced or abandoned. In this way, some 30% of mined P can be left in the ground (Drangert et al. 2018). Also, a vegetarian diet requires much less P compared to a meat- and milkbased diet, but it may be difficult to steer eaters away from meat. The above effective measures can significantly reduce the demand for virgin phosphate rock (Springmann et al. 2018). In addition, efficiency in the use of fertilisers in agriculture can be greatly improved.

We live in a chemical society with more than 140 000 man-made chemical substances in use, of which several thousands need to be investigated for both their acute and long-term effects (ECHA 2007; EU 2018). Authorities fight an uphill battle to control numerous new chemical products and compounds that households can purchase and dispose of in the sewer and trash bin. For instance, the EU Chemicals Agency (ECHA) leaves most of the evaluation of health and environmental risks to the industry itself according to rules outlined in the REACH regulation (ECHA 2007). Municipal utilities treating waste and wastewater could step in as whistle blowers. If people add fewer chemical compounds while using the water, the resulting sludge becomes easier to treat and sometimes harmless to recycle (Kümmerer 2007; Bergbäck and Jonsson 2008).

Since residents in a circular society know that the treated wastewater comes back to them in their taps, they are likely to be more careful with what they mix into the water while using it. Therefore, they may select cleaning products made of biodegradable or fast degrading chemical compounds in, e.g. soaps, washing powders and shampoos (Kümmerer 2007). For instance, greywater from dishwashing with biodegradable detergents can be used directly to water the garden. Not used pharmaceuticals and other toxic products are less harmful if disposed of in the solid waste stream and not flushed down the toilet. Government chemical agencies could assist laymen by requesting manufacturers to put easy-to-understand labels about toxicity of their products. With such information, the quality of the raw wastewater entering a mini sewage treatment plant (mini-STP) is likely to be markedly better than that received today at large municipal wastewater treatment plants and therefore easier to treat.

A potent alternative measure to enhance management of material flows is to avoid mixing all kinds of wastewater and avoid mixing all solid waste. Already, many cities have enacted by-laws that require pre-treatment of industrial wastewater before allowing its effluent to enter the communal sewer or surface waters. Likewise, polluted stormwater is ideally channelled through dedicated sewers or drains in order to prevent overflows of wastewater treatment plants during heavy rains. In the case of nutrients, these are concentrated to excreta and organic waste flows, and separate collection of biowaste and separate blackwater sewers can secure a safe and well-composed fertiliser containing all macro- and micro-nutrients as well as organic matter and few harmful compounds (Tervahauta, et al. 2014). Yet, the European Union (EU) regulations do not recognise neither blackwater nor excreta as separate entities but only as part of wastewater sludge. Today’s avoidance to separately circulate all nutrients in human excreta is likely to be due to cultural perceptions rather than a scientific distinction or technical challenge (Drangert et al. 2018).

An extra separate sewer for toilet water is cheap to instal in new houses, new cities and neighbourhoods, and this allows easy collection of most nutrients from households, and provides a nutrient-rich fraction with only insignificant unwanted chemicals. Segregated organic matter from household solid waste serves the same purpose of facilitating treatment and recycling of organic waste.

### Step 2: Optimise reuse of water and nutrients

Reuse means using the content of the various waste flows more or less as they are. Only a small part of the greywater from showers and bath tubs, washing machines, handwash basins, sinks, and floor cleaning can be reused directly due to chemical pollutants (Harder et al. 2018).

In this article, food waste and the nutrient-rich urine fraction are considered reusable (Step 2), while faecal matter is managed under the heading of recycling (Step 3) since it often requires some transformation before use. Urine contains most of the N and about half of P and K in human excreta, while faeces contain the other half of P and K, and almost all carbon (Rose et al. 2015). If collected separately, the almost sterile urine can be directly applied as an almost complete fertiliser in the family garden (WHO 2006). Otherwise, the urine can be treated by simple storage (WHO 2006) and used to fertilise public gardens, or be transported to neighbouring farms at acceptable environmental costs (Spångberg et al. 2014). The economic value of urine is not in the water fraction, but in its plant-available macro- and micro-nutrients (Vinnerås and Jönsson 2006). There is promising research to dehydrate urine so that only the nutrient powder is transported to the field (Senecal & Vinnerås 2017).

### Step 3: Optimise recycling of treated fractions

If the desired compounds in the wastewater and solid waste are not in a state that allows reuse, some kind of conversion into a new product is required. This step comprises two main options for wastewater sludge and organic solid waste: to treat to required quality or to keep fractions separate, or a combination of the two.

Almost all nutrients leaving a household originate from nutrient-rich excreta and food waste. For example, 80% of the P and 60% of the N discharged by European households are contained in the toilet water (Hellström et al. 2007). Kitchen waste together with so called blackwater, contain 80–92% of the nutrients N, P and K leaving households and can be kept separated and made available after some treatment (Zeeman and Kujawa-Roeleveld 2011). From a fertiliser perspective, the total content of N, P, and K in all toilet water in Sweden, before losses, amounts to 28, 44, and 55% respectively of the annually sold mineral fertilisers (Jönsson et al. 2012; Spångberg et al. 2014). Such high values are expected, since the human body essentially uses the energy in eaten food while almost all nutrients are excreted.

The blackwater from toilets can be treated by using ordinary treatment methods, e.g. the upflow anaerobic blanket (UASB) reactor that preserves the volatile N and saves on energy (Harder et al. 2018). Blackwater may also be mixed with the relatively nutrient-rich water from a kitchen garbage grinder. Pathogens can be inactivated without losing the nutrient value (WHO 2006; Strande et al. 2014). The storage time to hygienize faecal matter from urine-diverting dry toilets is some two years (WHO 2006), and this period can be reduced considerably by adding lime or urea (Fidjeland et al. 2013). Alternatively, earth worms and fly larvae can process faecal matter, dewatered blackwater, manure and organic waste into protein-rich animal feed (Lalander et al. 2013; van Huis et al. 2013).

Greywater represents the largest volume of used household water, and it may vary between, say, 20 to 200 lpcd and beyond (see Table 1), depending on the water-saving arrangements (Step 1a). The quality of this nutrient-poor water is affected by the kind of chemicals being used by residents. Man-made chemical substances often make the greywater sludge unfit for application on farmland (EC 2012), unless treated (Harder et al. 2018). However, greywater from environmentally concerned persons who essentially use biodegradable products can be treated in a simple adsorption filter or by an apartment-complex treatment plant. The resulting effluent quality is good enough to flush toilets, feed washing machines, clean floors and wash cars. Water for flushing of toilets and for irrigation, is in regular demand and only dual piping is required for this new water supply. In contrast, water for street cleaning, car wash, and firefighting has few quality restrictions, but the demand for it is low and sporadic.

### Steps 4 and 5: Incineration and landfilling of sludge and biowaste

Waste should ideally only be incinerated when all the valuable fractions have been removed in Steps 1–3. However, wanting sludge of recycling quality and prohibition to landfill sludge, make incineration attractive to municipal utilities and city councils (Kirchmann et al. 2017). Some countries, e.g. Switzerland, Germany, and several states in the U.S., have mandated incineration of sludge and recycling of P in the ash (de Boer et al. 2018). Austria’s new Waste Management Plan stipulates that, by 2030, P should be recovered from 65–85% of sewage sludge by incineration and recovery from ash or by precipitation of P from sludge or from P-rich effluents after sludge dewatering with a minimum recovery rate of 45% (OWAV 2018). In addition, sewage sludge ash will be registered as “non-hazardous” waste under a specific code number, and thereby reducing quality considerations.

But, the incineration option remains less sustainable since it removes all carbon, nitrogen, and sulphur which makes the ash less valuable for agricultural use. Huygens and Saveyn (2018) suggest that incinerated organic waste results in considerably lower agronomical efficiency than precipitated P salts and chemical fertilisers. Also, at temperatures above 800 °C, the amount of plant-available P in the ashes decreases (Zhang et al. 2001, 2002). Yet, some 30% of the wastewater sludge is incinerated in the EU member states, despite a policy of being circular societies (Milieu Ltd, WRC and RPA 2008; EC 2015).

Landfilling is restricted to materials and substances that are not managed under Steps 1–4. For example, Sweden report that only 1% of solid household waste is landfilled (Avfall Sverige 2018). Landfilled incinerated mixed material is likely to contain toxic substances that might dissipate or dissolve. Therefore, such toxic residues should be immobilised.

### Potential impact of measures in Steps 1–3 to recover water and plant nutrients

**Fig. 3:**
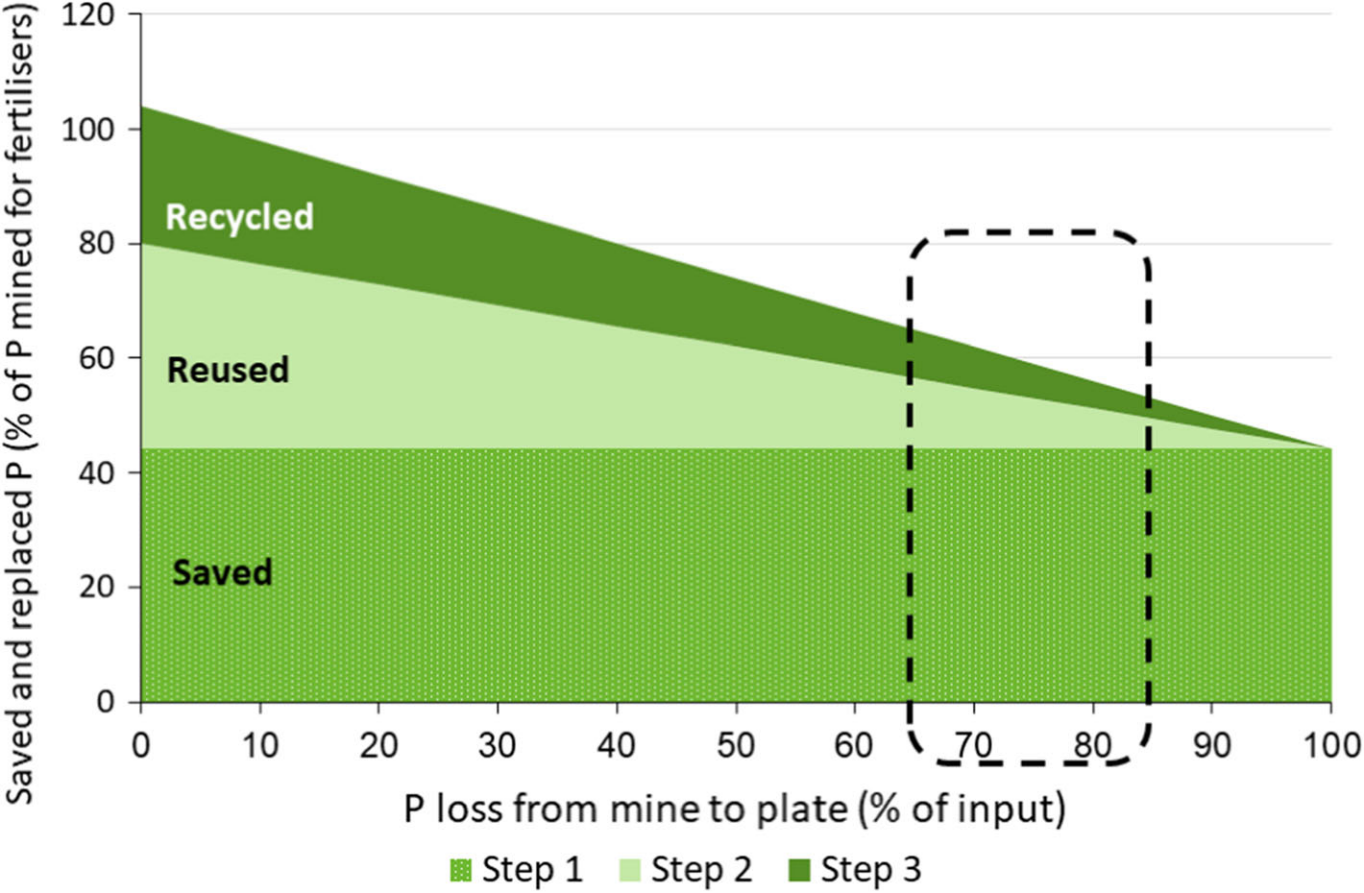
The demand for mined phosphorus (P) can be lowered by reducing food waste and food/feed additives, and by not using P in detergents (Step 1), and by replacing mined P in fertilisers with P in urine and food waste (Step 2), and with P in faeces and food waste (Step 3). The percentage losses from mine to plate impacts the proportion that can be replaced, as visualised by moving the dashed box that indicates the interval for such losses. *Source* Drangert et al. (2018)

Conditions differ vastly between cities and provide them with unequal opportunities to manage water and nutrient resources. Some quantitative data for Steps 1–3 of the extended waste hierarchy and the flexible water balance are summarised in Table 1 and Fig. 3.

The potential to reduce water consumption in cities is considerable. For example, modern shower heads save more than half the water, but shops continue to sell fancy and wasteful ones. Faucets can reduce water use by 85 up to 98% by fitting an adjustable nozzle suitable for most uses (Altered 2018). A modern dual-flush toilet uses only 0.3 L per urine flush and 3–4 L per big flush instead of some 10–13 L in older conventional toilets (Gleick 1993; Suzuki et al. 2010). A person urinates some 6 times per day and defecates only 1–2 times. Therefore, a dual-flush toilet can reduce the daily use of water by up to 65 lpcd compared to the conventional one. If a urinal or a urine-diverting toilet or vacuum toilet is installed the saving is even bigger. There are also odourless dry toilets on the market.

Washing machines and dish washers are today as water-efficient as washing manually: 6 L of water per kilogram clothes in comparison to the least efficient washing machine that used 14 L (How to save water 2018). In addition, there are washing machines which deionize the incoming water and, despite using only cold water and no detergents, they have the same washing effect as conventional machines (Råd and Rön 2018).

Today, most household water is used for personal hygiene, e.g. taking a shower and flushing the toilet. For example, a Swede uses on average 140 lpcd: 60 L for hygiene, 30 L for toilet flushing, 15 L for washing clothes, 15 L for washing dishes, 10 L for food and drink, and 10 L for other purposes (Svenskt Vatten 2017). A positive consequence of saving water is that the volume of wastewater goes down proportionally.

In summary, Table 1 shows the daily consumption in the U.S. of three to 400 L (Gleick 1993; WEF 2018) and 140 L in Sweden. Water-efficient devices can reduce the use by 55 L in Sweden. With modest reuse and recycling of greywater and some blackwater, the need for `imported´ fresh water is reduced further to 28 lpcd (140 L–55 L–12 L–45 L) as shown in column 5. A lower limit for household water usage is closer to a total of 70 litres than 140 lpcd or more.

Table 1 shows that much of the European Union farm need for mined P-fertiliser could be met by reduced demand for P by industry and reuse and recycling of household-derived P (Drangert et al. 2018). In Step 1, P use can be reduced by 30% and this amount, in turn, can replace over 40% of mined P presently used as fertilisers. A shift to more vegetarian food, could reduce this figure further by *x*%. In order to calculate the extent that reused and recycled P in excreta and food waste (Steps 2 and 3) can replace present-day application of P-fertiliser, we need to discount for losses from mine to plate. The dashed box in Fig. 3 estimates the range of current levels of losses to 65–85%, in which case savings in Steps 2 and 3 can replace another 15–30%. In total, the present demand for mined P can be reduced by half to two-thirds and thus prolong the lifetime of P deposits by several hundred years (Drangert et al. 2018). The situation is enhanced further if the nutrient efficiency in agriculture increases, as illustrated by a move to the left of the dashed box.

## Applying the flexible urban water balance—The Bangalore case

An illustration of the flexible water balance is from the Indian city of Bangalore of 9 million people (Drangert and Sharatchandra 2017). More than a century ago, an integrated water system, akin to the one in Singapore today except for technology, was gradually introduced in Bangalore as a chain of interconnected catchment areas with lakes and man-made tanks (High Court 2011). The then almost chemical-free local communities made it possible to draw water from these tanks for all household purposes, and it catered for a substantial part of the residents’ needs, while evaporation took its share. The system gradually deteriorated due to urban densification and encroaching illegal buildings and streets.

**Fig. 4:**
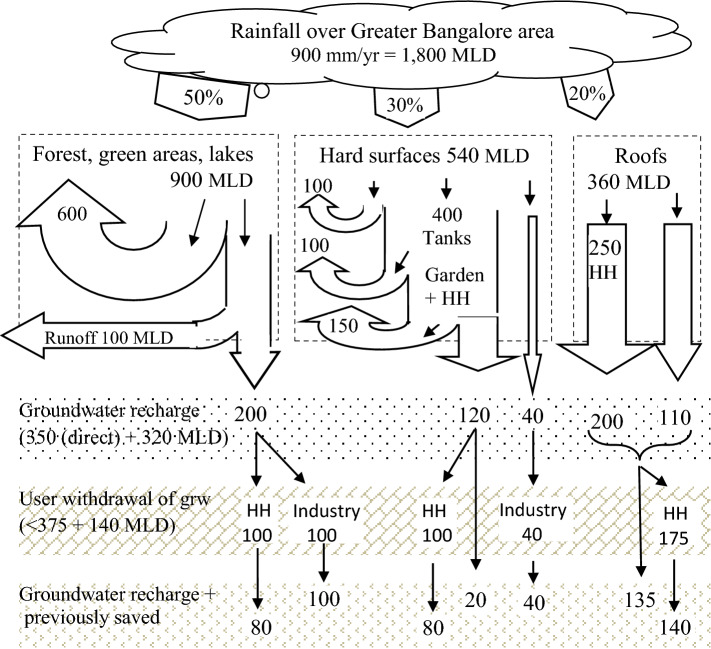
A ‘*flexible water balance*’ for rainfall over Greater Bangalore the year 2050 in MLD. Man can manipulate the rain over the three kinds of surfaces. After use once or more times the effluent recharges the soil and groundwater, and this water may be used again. *HH* households, *MLD* million litres per day. Arrows not to scale. *Source* Drangert and Sharatchandra (2017)

Today, the city’s water supply is pumped from the distant Cauvery river (1400 MLD) of which half is lost on route, and from the numerous city borewells (340 MLD) causing a serious overdraft of groundwater. The unit MLD (million litres per day) is here used throughout. The flexible water balance in Fig. 4 indicates that in the year 2050 when population may stand at 20 million, it is still possible to do without the river water, and to reverse depletion and instead recharge the groundwater, by collecting rainwater and carry out moderately ambitious Steps 1–3 for used water. Full-scale circular society arrangements such as the city-state Singapore show that this is possible.

Some 600 MLD of the 900 MLD rain over forests, green areas and lakes are taken up by trees and plants and transpired. Between zero and 300 (= 100 + 200) MLD of this rainfall can be made available from the runoff and infiltration, depending on arrangements ranging from none to what is being done in Singapore. 100 MLD of the 540 MLD falling over hard surfaces evaporate at once, and 40 MLD is infiltrated. 400 MLD are diverted to water tanks and treatment impoundments to be used mainly for irrigation and washing purposes, while after use 350 MLD evaportanspire or leak to soil. One-third, 160 (= 120 + 40) MLD, may enter soil and groundwater. Of the 360 MLD rain on roofs, 250 MLD are collected by households and, after reduction of 20% for consumptive use, altogether 310 (= 200 + 110) MLD enter the soil via recharge wells.

Households may collect the used water and use it again and again after appropriate treatment. Equation 1 shows the amounts of rainwater on the respective surfaces initially made available from man-made structures (impoundments, tanks, and sumps).

1 $$y \times \left( {1 + n_{1} } \right) \times 300\;{\text{MLD}} + \left( {1 + n_{2} } \right) \times 300\;{\text{MLD}} + \left( {1 + n_{3} } \right) \times 250\;{\text{MLD}}$$

where *n*_*x*_ = number of times of reuse/recycling, and *y* refers to the proportion (0 ≤ *y* ≤ 1) of MLD from green areas to infiltration and runoff that is being used by households.

A total of 670 MLD of fair-quality water is stored in the ground after deducting 20% for consumptive use: 350 MLD from direct infiltration and another 320 MLD from infiltration of reused and treated water. Theoretically, this added groundwater could be abstracted without lowering the saturated level. However, in this example only 515 MLD are withdrawn, 375 MLD and 140 MLD by households and industry respectively. Again, this fair-quality groundwater may be recycled once or more after use and treatment: n_4_ times by households and n_5_ times by industry. Equation 2 summarises the volumes of water available to users when drawing 515 MLD of replenished groundwater to be used by households and industry:

2 $$\left( {1 + n_{4} } \right) \times 375\;{\text{MLD}} + \left( {1 + n_{5} } \right) \times 140\;{\text{MLD}}$$

After deducting for consumptive household use, 440 (= 300 + 140) MLD is recharging groundwater and soil. In the whole process 595 (= 670 − 515 + 440) MLD have been added to the groundwater and soil. This is the essence of a ’flexible water balance’ in which various water sources are interacting.

The two equations offer policy-makers several options to combine the management tools at their disposal. For instance, a low rate of recycling where *y* = 0, *n*_1_ = 0, *n*_2_ = 0.5, *n*_3_ = 1, *n*_4_ = 1, and *n*_5_ = 2.5 provides 1325 MLD for households and 490 MLD for industries and still leave room for further reduction, reuse, and recycling in the future. This amount is enough to meet demands in the year 2050 for a total population of 20 million, up from 9 million, with a 70 lpcd water use. The disputed water from Cauvery river is not utilised in this case and the non-use of river water would ease political tensions downstream. Groundwater, instead of being mined as is presently the case, is substantially recharged and represents a huge unconfined storage potentially available for additional uses.

## Discussion

Urbanites can secure water and food supply while protecting human health and the environment by improving the eco-design upstream. Up to now, two main features have guided the urban plans for the water and solid waste sectors: large city-wide technical systems and low formal participation by residents. This is changing in our age of electronic communication and monitoring, where users are increasingly exposed to metering of water use and waste disposal. Feedback information could raise residents’ awareness of the value of prudent water usage, as could a timer in the shower head. Also, the challenges facing utilities in a chemical society have made them more willing to invite users to help ameliorate urban waste and pollution problems, foremost by switching to more biodegradable products and by segregating solid waste. A chip on each product in shops with information about the product’s effect on the performance of the utilities and impact on the health of humans and the environment would assist in creating informed buyers who are likely to purchase more environmentally friendly household products (detergents, shampoos, cleaning agents, etc.). Such transparency would also force manufacturers to abandon some toxic substances and go for less harmful products (Bergbäck and Jonsson 2008).

### Environmental foot prints at city level

Ecological housing projects are springing up worldwide. The designs range from greening a suburb to self-contained houses independent of utility services. The intention is to create a sense of “return to nature” in an urban setting. Three examples of eco-buildings are given.

**Fig. 5:**
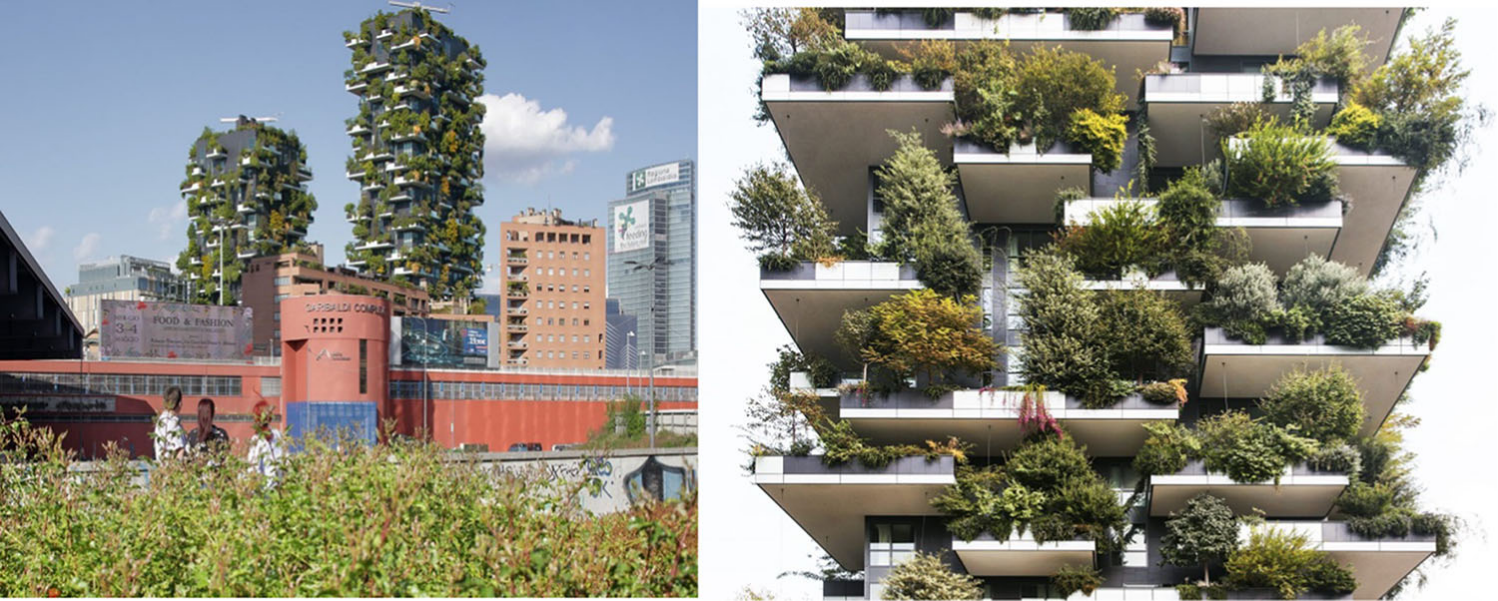
The Vertical Forest suburb El Bosco in Milan with 27-storey apartment buildings with planted balconies. *Source* Vertical Forest (2018)

A new suburb in Milan city in Italy has high-rise eco-buildings with big balconies for trees and other plants (Fig. 5). The residents can harvest fruits and berries and enjoy shade from the sun in the summer. No air conditioners are needed, while some leaves fall off in the winter and allows the sun to heat apartment walls. The plants provide a humid micro-climate, absorb carbon dioxide and dust particles, and produce oxygen. The total balcony area is several times larger than the space occupied by the house itself, and the flat land required for these plants and trees would cover an area of 10 000 m^2^ (Vertical Forest 2018).

**Fig. 6:**
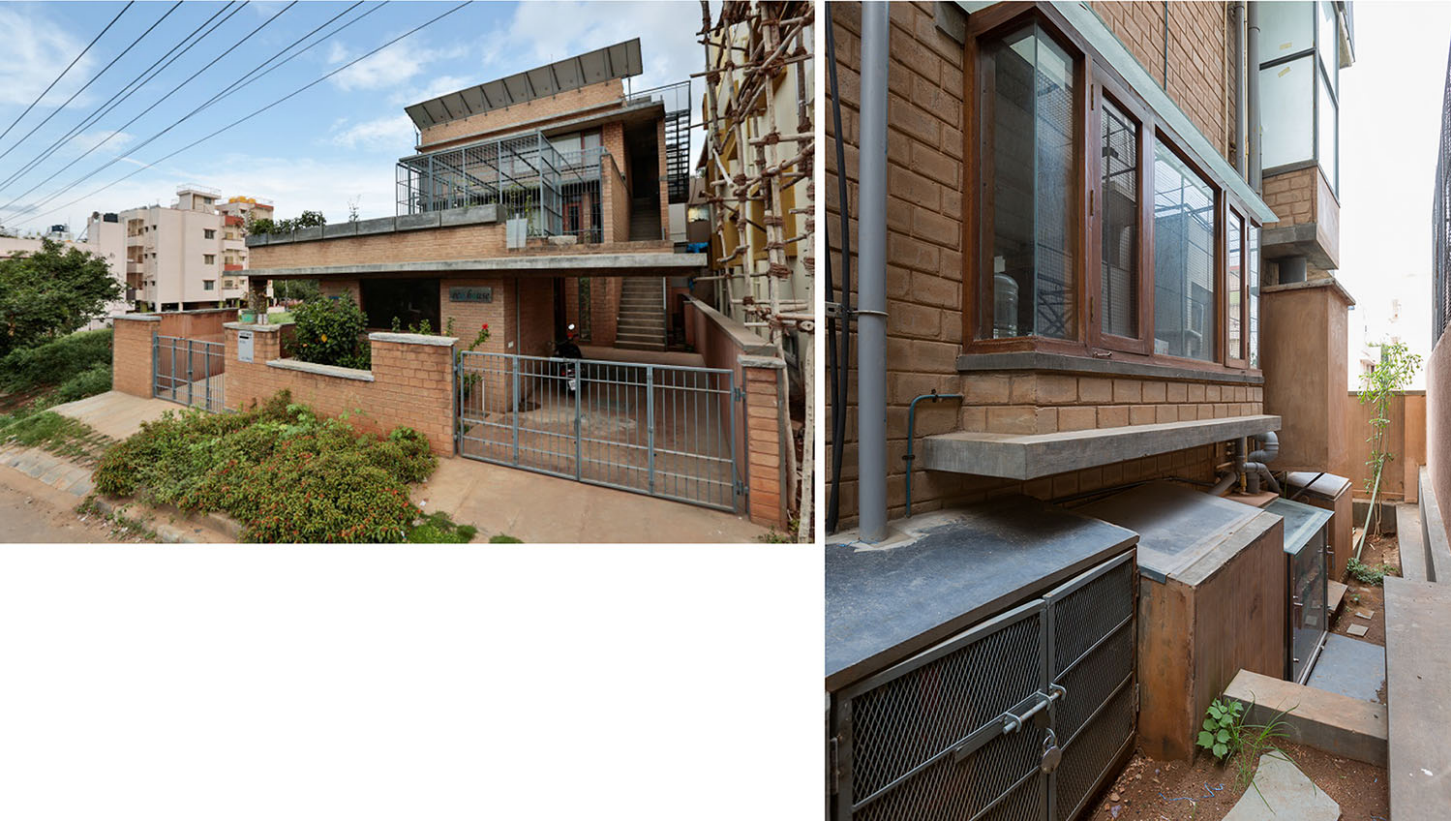
The ecohouse with solar panels and gated gardens (left), and the treatment units at the back of the house (right). *Source* Ecohouse (2014)

An extended-family house in India (Fig. 6) has been designed to recover and use water and nutrients in situ, while the sun produces all energy (Ecohouse 2014). This 3-storey ecohouse on a plot of 240 m^2^ avails 60 m^2^ of roof area to rain catchment and 100 m^2^ for plants. Rainwater is stored in an underground sump and used for drinking, shower bath and food preparation, while recycled greywater is used for flushing low-flush toilets, filling the washing machines, and for watering plants and cleaning floors. Urine and faecal matter are collected separately, treated and used to fertilise plants and trees. All treatment units occupy a small area at the back of the house. The bathrooms and kitchens are placed towards this back wall in order to minimise piping and secure vertical flows to avoid blockages.

**Fig. 7:**
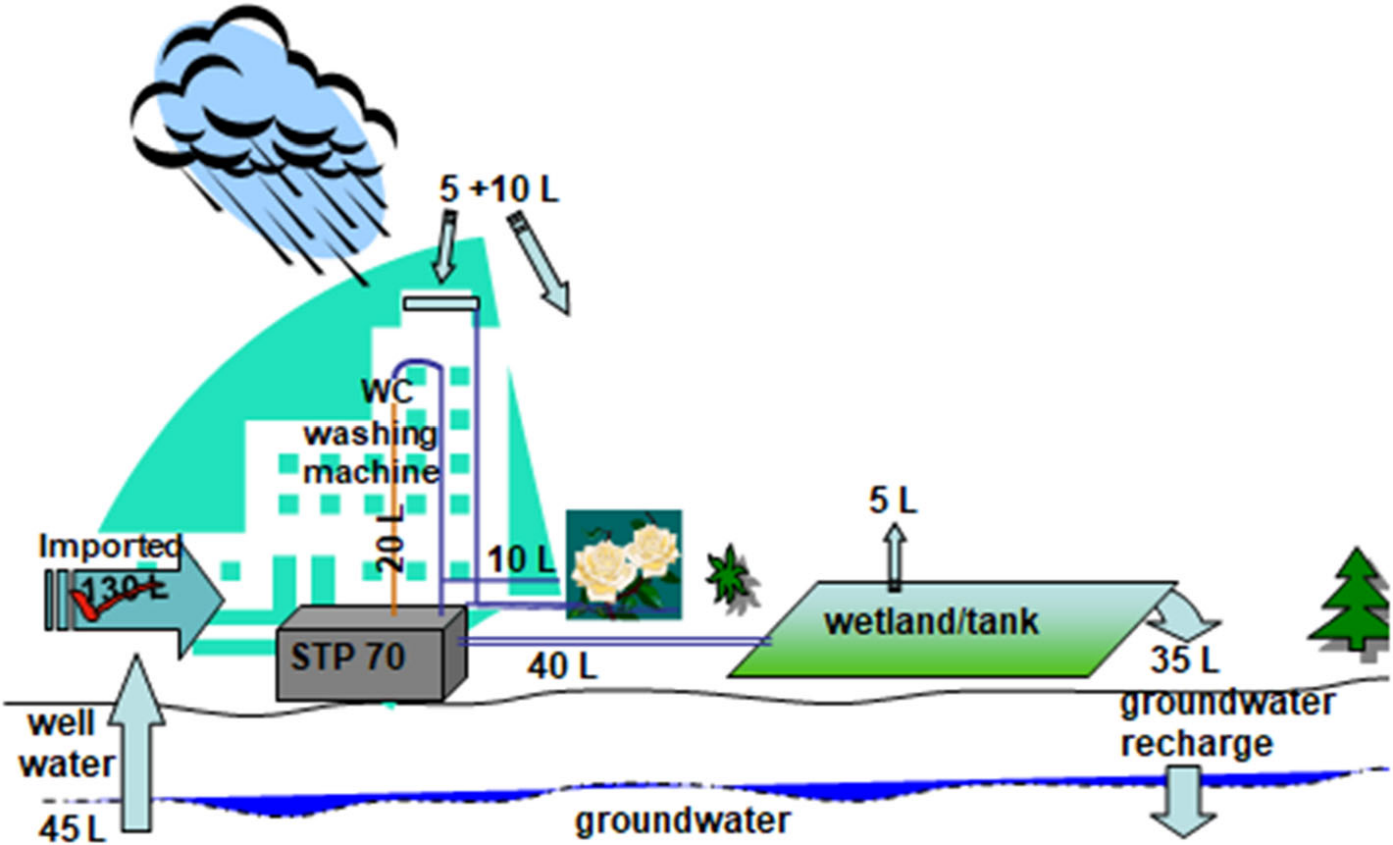
A self-contained housing complex with rainwater catchment and separate local treatment of grey- and black water, and reuse/recycling of effluent water and nutrients

The generic example in Fig. 7 illustrates a water- and nutrient-efficient design for an eco-complex with apartments. All rain is catched and no water is needed from the municipal utility. No discharges are left unmanaged, and the treated water and plant nutrients are recirculated locally.

Residents use 70 lpcd on average (after Step 1a), and each flat has dual water supply pipes, one pipe to flush toilets and fill washing machines, and one for drinking water and all other purposes. Also, there is dual wastewater piping for discharges, one for toilet water and the other for greywater. The two treatment plants for black water and greywater are complementary and deliver: 20 lpcd to flush toilets and fill washing machines, 10 lpcd of treated effluent to water gardens, and the remaining 40 lpcd of treated greywater is either used straight away or polished in a wetland before use. Some direct evaporation, say, 5 lpcd from the wetland and 10 lpcd rainfall on the garden allow for a total of 45 lpcd of forced infiltration. Thus, 45 lpcd of good-quality water can be drawn from a sump or groundwater wells without affecting the groundwater level. In order to ensure drinking water quality, this water can be treated in a purifier before delivered to the flats. If the use of harmful chemical products is reduced enough, the addition of rainwater is enough to keep the accumulation of chemicals down. Together with 5 lpcd recycled roof-catchment water and the 20 lpcd treated recycled water, a resident is assured access to 70 lpcd—without any import of water to the eco-complex.

The nutrients in the blackwater pipe (or in the urine and faecal matter) is caught as good-quality sludge that can be treated and applied in gardens or on farmland, possibly co-composted with other organic solid waste from the kitchen. The little sludge arising from greywater treatment, on the other hand, may be dewatered and incinerated or landfilled.

The above examples of eco-designed houses and infrastructure are transparent and give feedback about effects of both good and poor resident behaviour. A higher awareness of harmful pathogens and toxic chemicals is likely to promote public participation in a sustainable management of the urban water and nutrient flows.

### Opportunities to guide urban planning

Cities have a unique opportunity now to embark on the above measures to secure supply of water for urban residents and nutrients for food production. In this century, the urban world population will increase from 3 to an estimated 8.5 thousand million (OECD 2013). This means that twice the number of dwellings and offices that existed in the year 2000 are to be erected during this century. All new buildings could be made water-smart and catch and store rain, collect blackwater in separate sewers, recycle used water and nutrients, and be fitted with water-saving appliances and fixtures. At the end of the century, at least two-thirds of all buildings are water- and nutrient-efficient—without additional costs for retrofitting. The already existing houses have to be retrofitted during this century and therefore these houses can also be made water- and nutrient-efficient with moderate extra investment. The result is that the urban population, as shown in Singapore, will have access to enough safe water in the foreseeable future and farmers will have access to sufficient plant fertilisers.

Space is required for all city activities, and urban history tells us that a new need can radically transform an existing urban layout. A striking example is the prominence given to vehicles in city planning in the last one hundred years. Buildings were pulled down to widen streets and generous regulations were introduced for, e.g. generous parking space per household. Novel environmental foot prints caused by vehicles, such as air and noise pollution were accepted as unavoidable. But, congested cities are now under pressure to reducing such environmental problems and alternative solutions for transport needs are being developed. Of late, electric bicycles and scooters are becoming popular in big cities. The ongoing re-evaluation of urban transport systems argues for an increased priority for the use of high-occupancy vehicles (Suzuki et al. 2010) and pedestrian and bicycle lanes.

**Fig. 8:**
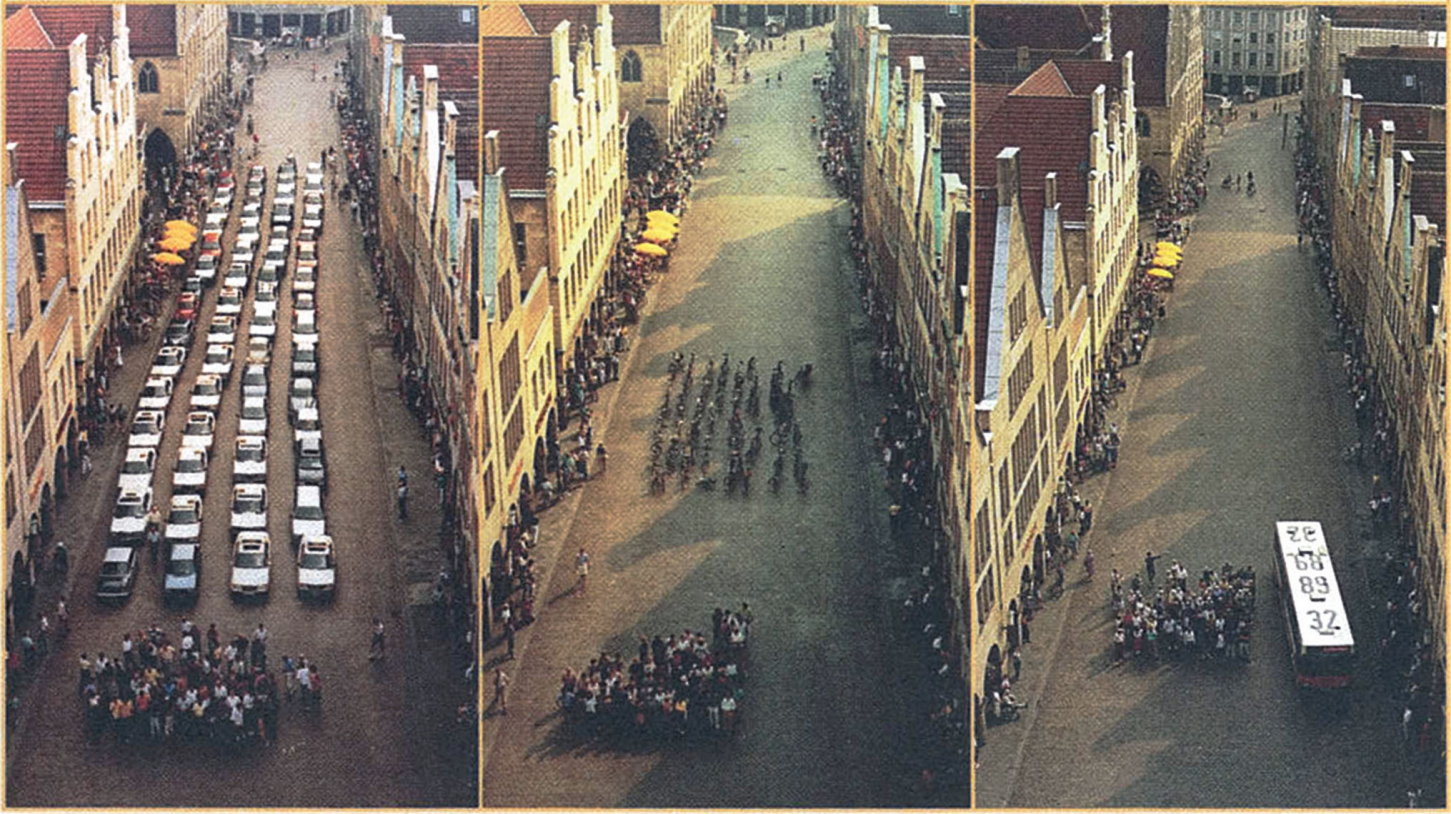
Roadway required by the same passengers travelling by car, bicycle, or bus, and alternative greening of the freed city space. *Source* Adapted from Petersen and Wuppertal Institute (2004) as reproduced in Suzuki et al. (2010)

Figure 8 indicates that, if wisely designed, substantial city space could be freed and converted into green areas and give room to collect rainwater, recycle treated wastewater sludge and organic matter, recycle effluent and/or infiltrate it into soil and groundwater.

## Conclusion

The unprecedented growth in world population results in overuse of virgin water and global nutrient resources, while urbanisation and increased human consumption cause huge amounts of waste locally. A gradual shift away from purposeless urban discharges of wastewater and nutrients can, again, strengthen the link between urban sanitation and agriculture. Sustainability requires a rethink of urban management of not least wastewater and biowaste and conversion of today’s linear systems into circular ones.

There is rarely an unavoidable shortage of water in urban areas or nutrient shortage in agriculture. Such limited supply is likely to be due to poor city design or planning and management of the available water and nutrient resources in urban areas. Urban infrastructure and buildings in water- and nutrient-efficient cities are technically fairly simple, and the main new item is a separate sewer for black water or urine. Only modest modifications of residents’ routines are required to operate the smart fixtures and devices, while water professionals, plumbers and farmers are to adjust to additional new resources concerns when applying their knowledge and skills. A circular system is to a large extent a mental change, away from thinking in terms of abundant global resources and sinks, to realising that the globe has finite resources and space. Close to zero import of virgin water to urban areas and export of most household nutrients back to agriculture is within reach and should become the aspired goals in urban design and planning. This will pave the way to achieving the UN Development Goals 2030 for these two sectors.
