# Correction to: Urban water and food security in this century and beyond: Resource-smart cities and residents

**DOI:** 10.1007/s13280-020-01480-z

**Published:** 2021-01-16

**Authors:** Jan-Olof Drangert

**Affiliations:** Department of Water and Environmental Studies, Linköping University, Linköping, Sweden

## Correction to: Ambio 10.1007/s13280-020-01373-1

In the original publication, Table 1 was processed incorrectly during the typesetting and publication process. The final column of Washing clothes should be “−10” and not “−1” in Table 1. The correct version of Table 1 is provided in this correction.

**Table 1:** Various measures by urban households in some countries to reduce, reuse and recycle water in litres per person per day and nutrients as percentage of imported mined P. Sources: Gleick (1993), Suzuki et al. (2010), Svenskt Vatten (2017), Drangert et al. (2018)

Measures to save H_2_O in some rich cities/countries					Measures to save P in EU member states	
	USA ca 1990 Range	USA Least lpcd	Sweden today (L)	Aim in lpcd (L)		EU import (%)
**Step 1: reduce**						
Hygiene eg. shower	20–30 (L per min)	100–150 L 5 min/day	60	− 30	No P in detergents	− 6
Toilet flush	10–30 (L per flush)	75–210 L 7 times/day	30	− 15	No P in additives	− 14
Washing clothes	150–210 (L per load)	35–52 L once/week	15	− 5	Less food waste	− 10
Washing dishes	50–120 (L per load)	17–40 L twice/week	15	− 5	More vegetarian food	− *X*
Prepare food + drink	No measure	20 L	10	0		
Other	No measure	20 L	10	0		
***Subtotal***		***267–492 L***	***140***	− ***55***		**>** − ***30***
**Step 2: reuse**						
Greywater	0	0	0	− 10	Urine	− 31
Blackwater	0	0	0	− 2	Food waste	− 5
***Subtotal***	***0***	***0***	***0***	***− 12***		***− 36***
**Step 3: recycle**						
Greywater	0	0	0	− 35	Faeces	− 16
Blackwater	0	0	0	− 10	Food waste	− 8
***Subtotal***	***0***	***0 ***	***0***	***− 45***		***− 24***

The original article has been corrected.

